# Improving cardiovascular control in a hypertensive population in primary care. Results from a staff training intervention

**DOI:** 10.1080/02813432.2024.2326470

**Published:** 2024-03-08

**Authors:** Rebecka Quester, Staffan Björck, Karin Manhem, Jonatan Nåtman, Susanne Andersson, Per Hjerpe

**Affiliations:** a Närhälsan Bollebygd Health Care Centre, Bollebygd, Sweden; b Primary Health Care, School of Public Health and Community Medicine, Institute of Medicine, Sahlgrenska Academy, University of Gothenburg, Gothenburg, Sweden; c Centre of Registers Västra Götaland, Gothenburg, Sweden; d Department of Molecular and Clinical Medicine, Institute of Medicine, Sahlgrenska Academy, University of Gothenburg, Sweden; e Department of Emergency Medicine, Sahlgrenska University Hospital, Gothenburg, Sweden; f Regionhälsan R&D Centre, Skaraborg Primary Care, Skövde, Sweden

**Keywords:** blood pressure, hypertension, primary healthcare, intervention, nurse

## Abstract

**Objective:**

A pilot study to evaluate a staff training intervention implementing a nurse-led hypertension care model.

**Design and setting:**

Clinical and laboratory data from all primary care centres (PCCs) in the Swedish region Västra Götaland (VGR), retrieved from regional registers. Intervention started 2018 in 11 PCCs. A total of 190 PCCs served as controls. Change from baseline was assessed 2 years after start of intervention.

**Intervention:**

Training of selected personnel, primarily in drug choice, team-based care, measurement techniques, and use of standardized medical treatment protocols.

**Patients:**

Hypertensive patients without diabetes or ischemic heart disease were included. The intervention and control groups contained approximately 10,000 and 145,000 individuals, respectively.

**Main outcome measures:**

Blood pressure (BP) <140/90 mmHg, LDL-cholesterol (LDL-C) <3.0 mmol/L, BP ending on −0 mmHg (digit preference, an indirect sign of manual measuring technique), choice of antihypertensive drugs, cholesterol lowering therapy and attendance patterns were measured.

**Results:**

In the intervention group, the percentage of patients reaching the BP target did not change significantly, 56%–61% (control 50%–52%), non-significant. However, the percentage of patients with LDL-*C* < 3.0 mmol/L increased from 34%–40% (control 36%–36%), *p =* .043, and digit preference decreased, 39%−27% (control 41%−35%), *p* = 0.000. The number of antihypertensive drugs was constant, 1.63 − 1.64 (control 1.62 − 1.62), non-significant, but drug choice changed in line with recommendations.

**Conclusion:**

Although this primary care intervention based on staff training failed to improve BP control, it resulted in improved cardiovascular control by improved cholesterol lowering treatment.

## KEY POINTS

- Hypertension is common and often suboptimally treated in relation to existing guidelines.
- This register study evaluates the results of a staff training intervention promoting nurse-led care.
- The intervention had an impact on measurement techniques, drug choice and improved cholesterol control.

## Introduction

High blood pressure is a leading modifiable cause of premature death [1]. In Sweden, approximately, a third of the grown-up population is affected [2,3]. Many patients with hypertension do not reach recommended blood pressure targets and are thereby exposed to an unnecessary – but treatable – elevated risk of disease and death [4].

The Västra Götaland region (VGR), in the south-west of Sweden, has a mixed urban and rural population of 1.7 million inhabitants [5]. About 200 primary healthcare centres (PCCs) treat approximately 170,000 patients with uncomplicated hypertension (without diabetes or coronary heart disease). According to the regional primary care quality register (QregPV), only half of these patients (56%) reach the recommended blood pressure target <140/90 mmHg, varying between PCCs from 25 to 85% [6]. The proportion of patients achieving the recommended low-density lipoprotein cholesterol (LDL-C) target <3.0 mmol/L [7] varies from 25% to 65% [6].

Antihypertensive treatment routines and visit patterns also vary. To what extent, and in which ways, this affects the ability to achieve treatment targets is yet only partially explored. However, previous studies in VGR have shown that nurse-led hypertension care is associated with better treatment control of blood pressure [8] and a lower incidence of stroke [9]. Successful improvement strategies for hypertension management often include task shifting and teamwork [10]. Clinical inertia among physicians is also a known factor causing suboptimal blood pressure control, which can be reduced by interventions focusing on education, written memos, reminders, and evaluation [11,12].

In the light of these facts, an intervention to further improve and standardize antihypertensive treatment in VGR in the form of a pilot study was initiated 2018. A new hypertension care strategy focused on training of personnel, including an increased responsibility for the nurses in the diagnosis and management of the patients, was implemented [13].

The aim of the study was to describe the effects of this intervention on the achievement of treatment goals for blood pressure and cholesterol levels.

## Methods

### Study design

An observational regional register study on the achievement of reaching treatment goals for blood pressure and cholesterol in hypertensive patients without major comorbidities after implementation of a new model of care in primary care. All PCCs in VGR not participating in the intervention were used as controls.

### Intervention

All 202 PCCs in VGR were invited to the intervention and 12 were accepted. From each included PCC, 1–3 nurses and/or physicians participated in five whole-day seminars. The basic idea was to update and advance knowledge and provide standardized protocols for the treatment of hypertension including a task shift from physician to nurse. In this protocol, the nurse responsibility was extended from the regular check-ups to include diagnosing, risk stratification and drug titration under the supervision of the responsible physician, who prescribed the recommended drugs [13].

The protocol described the division of responsibilities between physician and nurse regarding examination procedure and assessment of risk factors. A step-by-step recommendation of antihypertensive and cholesterol lowering medication titration, and follow up was also included, supplemented with checklists concerning hypertension resistant to treatment, side effects, and recommended lifestyle changes. The participants were encouraged to survey their current local work procedures, adapt the recommended routines to local circumstances, and finally implementing them. On the seminars, the participants were able to discuss their progress, if there were any difficulties with performing the task shift as well as questions about the protocol, complicated cases and how to interpret the national recommendations. The intervention’s protocol recommended, in short [13]:

- Nurse-led care
- Digital blood pressure monitors
- Blood pressure target <140/90 mmHg
- LDL-C target <3.0 mmol/L in general after individual risk evaluation
- Multiple drug therapy preferred before increase in doses
- Antihypertensive drugs, recommended substance and order: 1. Candesartan, 2. Bendroflumethiazide, 3. Amlodipine.
- Cholesterol lowering drugs, recommended substance and order: 1. Atorvastatin, 2. Rosuvastatin and/or Ezetimibe

The protocol originated from a successful local quality improvement effort a few years earlier at one of the region’s PCCs. It recommended a fixed drug choice in order to facilitate the drug choice process, based on then current regional recommendations for first choice substances [14]. The protocol chose angiotensin receptor blockers (ARBs) over ACE inhibitors (ACEis) to facilitate the handling of patients by lowering the risk of side effects. However, it was not recommended to terminate already initiated treatments with other substances (such as ACEis). It is worth noting that updated international treatment recommendations have been presented in 2023 [15].

### Study population

The study population consists of all individuals with hypertension but without diabetes or coronary heart disease, registered in QregPV at two time periods, before (1 June 2018) and after (1 September 2020) the implementation of the intervention.

One of the 12 initially included PCCs dropped out at an early stage. Patients from this PCC have been excluded in the analysis. The remaining eleven included PCCs had in all 10,534 patients with hypertension registered at baseline. The mean size of the PCCs (total number of patients enrolled at each PCC) was 10,352 (varying from 4,823 to 17,889). The 190 control PCCs had 145,363 hypertensive patients registered, with a mean total PCC size of 8,337 (varying from 455 to 19,177).

### Databases

The baseline and follow up information was based on data obtained from linking three regional registers through the patient’s unique personal identification number.

- QregPV, a regional primary care quality register including all individuals in VGR with a diagnosis of hypertension, coronary heart disease or diabetes registered in primary care medical records (private and public). Each month data on latest registered blood pressure, LDL-C and PCC enrolment are automatically collected for the included patients [16–18].
- Vega, a regional administrative health care database, covering all health care visits in VGR. It collects data on registered diagnoses, dates of visits and staff categories [19,20].
- The Regional Prescribed Drug Register Digitalis, containing information on all dispensed prescriptions [21].

### Statistical methods

For both assessment points, medical data were collected for each patient in accordance with the last observation carried forward method. The latest entry of each variable was used up to 450 (900 for LDL-C) days before the assessment date [17]. Medication profiles for each patient was based on drug prescription data for 120 days preceding the assessment points. Change in number of physical visits to the health centre were evaluated in the year before 1 March 2020 to avoid the impact of the pandemic on the visit pattern.

The difference-in-difference estimator was used to analyse the changes in outcomes for the intervention and control units. We used linear ordinary least square regression models to estimate the difference-in-difference interaction coefficients. Cluster robust standard errors were used to account for intra-class correlation within the units [22]. *P*-values <.05 were considered statistically significant.

## Results

The mean age of patients with hypertension in both groups was 69 years and 55% (intervention) and 56% (control) were male.

In Table 1, the results in blood pressure and cholesterol data at baseline (2018) and after 2 years (2020) are presented. There was no significant difference in change in blood pressure or percentage of patients achieving blood pressure treatment target. However, LDL-C decreased significantly more in the intervention group and the percentage of patients with LDL-*C* < 3.0 mmol/l increased. Digit preference decreased in both groups, but the decrease was twice as large in the intervention group.

**Table 1.** Blood pressure, LDL-cholesterol and visits per year.

	2018		2020		Difference		Significance
Year	Intervention	Control	Intervention	Control	Intervention	Control	*p* diff(I) – diff(C)
Blood pressure:							
Systolic (mmHg)	135.70	137.44	135.34	137.35	−0.37	−0.09	0.680
Diastolic (mmHg)	79.97	80.55	79.63	80.69	−0.34	+0.14	0.447
<140/90 (%)	56.27	49.61	60.91	51.95	+4.64	+2.34	0.362
> =150 systolic (%)	14.08	18.20	15.14	18.55	+1.07	+0.36	0.840
Cholesterol: LDL-C (mmol/L)	3.38	3.36	3.27	3.37	−0.11	+0.01	0.032
<3.0 mmol/L (%)	34.25	35.60	39.84	36.06	+5.59	+0.46	0.043
Digit preference:							
Blood pressure value ends with 0 (%)	38.80	41.15	26.71	34.93	−12.09	−6.22	0.000
Visits per year:							
Nurse	0.85	0.66	0.84	0.59	−0.00	−0.08	0.498
Physician	0.82	1.02	0.73	0.95	−0.09	−0.06	0.533
Ratio nurse/physician	1.04	0.72	1.16	0.70	+0.11	−0.02	0.559

Descriptive data including blood pressure, LDL-cholesterol and visits per year at baseline (2018) and two years later (2020) for intervention group and control group. Change from 2018 to 2020. Significance for the difference in change from baseline between the intervention and control groups.

The mean annual number of visits to a nurse or physician for patients with hypertension varied greatly between different PCCs in the intervention group (from 1.21 to 2.36) but with a similar mean at baseline (1.67) as the control group (1.68). There was a significant decline in visits in the whole hypertensive cohort (patients from all PCCs in intervention and control put together) in 2020 from 1.68 to 1.55 (*p* < .001), but no significant difference in change from baseline between the intervention and control groups. There was a trend of increase in the ratio nurse/physician in the intervention group and decline in the control group, but the difference in change from baseline was nonsignificant. We noted a minor increase in total number of other contacts than physical visits (telephone contacts etc.) in the whole hypertensive cohort, from 0.93 to 0.99 (*p* < .001).

**Figure 1.**
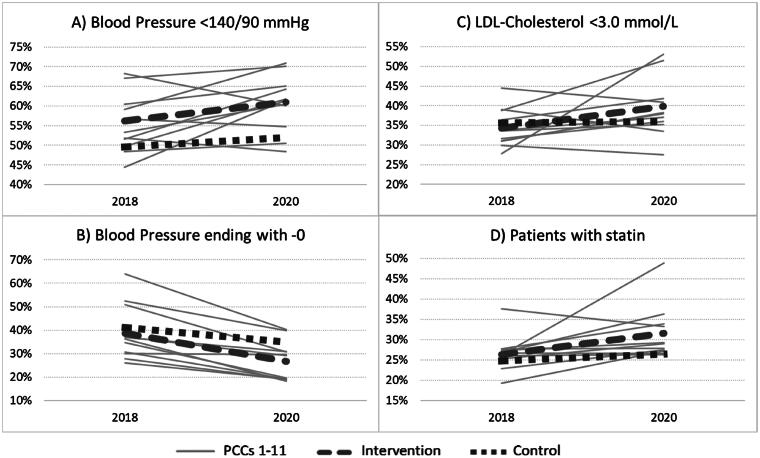
Separate results for the 11 PCCs included in the intervention (PCCs 1-11), their mean (Intervention) and mean for control group (Control). Progress from 2018 to 2020 (X-axis) in percentage of hypertensive patients (Y-axis) reaching blood pressure target <140/90 (upper left, A), with blood pressure value ending on -0 (digit preference, down left, B), reaching LDL-C <3.0 mmol/L (upper right, C) and with statin (down right, D).

Figure 1 presents the results for each separate PCC included in the intervention. The blood pressure results showed a wide range (<140/90 target fulfilment 45% to 70%) in a scattered pattern whereas digit preference results showed a uniform pattern of decrease. In a majority of the PCCs, there was an increase in the percentage of hypertensive patients treated with statins, although this did not reach statistical significance (*p* = .07).

Table 2 shows compiled data of drug prescription. In both the intervention and the control group the use of ACEis decreased while the use of ARBs increased. The transformation was more explicit in the intervention group, with a large increase of the intervention’s first-hand substance recommendation, candesartan. The use of beta blockers (BBs), not recommended in the intervention, decreased significantly more in the intervention group.

**Table 2.** Dispensed antihypertensive drugs and statins.

	2018		2020		Difference		Significance
Year	Intervention	Control	Intervention	Control	Intervention	Control	*p* diff(I) – diff(C)
No of BP drugs	1.632	1.615	1.636	1.624	+0.004	+0.008	0.824
ACEi (%)	23.58	24.06	19.85	22.24	−3.73	−1.82	0.002
ARBs (%)	39.20	35.61	46.00	40.14	+6.80	+4.53	0.033
candesartan (% of ARBs)	34.17	28.50	53.70	38.20	+19.53	+9.70	0.000
CCBs (%)	34.33	36.08	35.29	38.04	+0.96	+1.95	0.364
amlodipin (% of CCBs)	67.62	60.49	70.69	63.72	+3.07	+3.22	0.518
Diuretics (%)	25.77	25.31	24.37	22.87	−1.41	−2.44	0.214
BBs (%)	30.81	30.69	28.37	29.40	−2.45	−1.29	0.035
Statins (%)	26.37	24.76	31.54	26.46	+5.17	+1.69	0.071

Descriptive data for dispensed drugs. Mean number of antihypertensive drugs dispensed to hypertensive patients and percentage of hypertensive patients with different types of antihypertensive drugs and statins in intervention group and control group, respectively. Change from 2018 to 2020. Significance for the difference in change from baseline between the intervention and control groups.

## Discussion

### Statement of principal findings

The study failed to show an effect on blood pressure control. However, LDL-C levels were lowered in the intervention group, and there was a marked decrease of digit preference. There was also a change in choice of drugs in line with the intervention’s recommendation, although the total number of antihypertensive drugs did not change. The analysis of visit patterns showed higher nurse/physician ratio at baseline in the intervention group that possibly increased further, but not significantly.

### Strengths and weaknesses of the study

A strength with the study is that it included all hypertensive patients without diabetes or coronary disease in VGR, and that the data were collected from a real-life everyday clinical setting *via* regional registers.

A weakness is the selection of participating PCCs, as there was no randomization but based on individual choice from each PCC. Invitation to participate was presented to all PCCs in the region, and the 11 included PCCs were the ones that chose to sign up, whereas the controls did not respond to the invitation. We can assume that the participating PCCs were more interested and susceptible to improving their hypertension care than the non-responders. At baseline, there was a higher nurse/physician ratio at the participating PCCs, which could imply that nurses handled more of the hypertension care already before the intervention. The populations were not comparable as randomized groups and, therefore, we focused on change and not absolute numbers in the results. The short follow-up time should also be considered a weakness.

An aspect that possibly affected our results was the Covid 19-pandemic’s restrictions that hit the region in the latter part of the studied period. Probably both participants and non-participants were affected to a similar extent.

### Findings in relation to other studies

There are several examples of interventions with positive effects on hypertension management, and identified keys to success are combining education, counselling and management strategies [23], task shifting and teamwork [9,10]. However, the implementation of interventions in the everyday clinical setting is challenging [24], and the detectable changes in blood pressure on population level are small: A meta-analysis of interventions on hypertension between 2008 and 2018 [23] showed that the most effective interventions detected a difference in change in blood pressure on a level of −5.34 mmHg (systolic)/ −3.23 mmHg (diastolic), through combining strategies focused on lifestyle changes with strategies aimed at adherence to medication and monitoring blood pressure, cholesterol levels, etc.

A Finnish study, in 2014 [25], illustrated how an intervention shown to be effective in a tightly controlled research setting failed to improve blood pressure control when implemented in a real primary care setting. Lack of motivation and incentives among the staff was pointed out as a contributing factor to this lack of effect. A Swedish study in 2019 [26] stated that a simple audit and feedback intervention directed toward physicians in primary care did not improve secondary prevention medication for ischemic stroke/TIA patients, and suggested a more profound approach to the intervention (repeated reminders, wider recipients, graphic appeal) for possible success. Another Swedish study of an intervention in the county of Västerbotten 2020, educating physicians and giving feedback, managed to show positive results concerning blood pressure levels [12], though a follow-up study 2022 looking at hard end points (stroke, heart failure) failed to show a significant change [27]. As it was an observational study of a health care intervention, and not an actual clinical trial, there were possibly unknown factors (such as country of birth, interference of other interventions and cholesterol levels) also contributing to the outcome.

Even if the intervention in our study was composed of known strategies for possible success and the intervention PCCs were a selected group that had expressed interest, the results showed no significant improvement in blood pressure control. The decrease of digit preference in the intervention group is an indirect sign of increased use of automatic blood pressure monitors, a measuring technique considered more accurate and non-biased than manual measuring. The increase of automatic measures could mean fewer blood pressures close to the cut-off value being rounded down, a known phenomenon described in earlier studies that decreases when automatic measures are used [28, 29]. This could affect the proportion of patients achieving target blood pressure, but it seems to have little importance for the mean blood pressure, as shown in previous results from the region [30]. Our results did not show significant difference in effect on mean blood pressure, nor in percentage of patients with higher systolic blood pressure (>150 mmHg), between the groups. This is also in line with the absent change in number of antihypertensive drugs used in both groups, which is somewhat surprising, as one of the main recommendations in the intervention was to add more medications if the blood pressure target was not met.

The accuracy of blood pressures readings collected from health care records has an undesirable variability when used for scientific purposes [31], mixing office blood pressure with home measuring, acute blood pressures etc. Though blood pressure measuring is considered to be one of the most basic procedures in health care, it is challenging to achieve accurate and reliable values. Standardized measurement procedures are often neglected in the clinical practice and both falsely high and low values are common [31]. We see a large variation between PCCs when it comes to target fulfilment [6], and local routines as well as individual caregivers can vary, both in treating the patients and in measuring and documenting blood pressures. To take local differences into account we used a cluster model for statistical calculation which influenced statistical significance on several comparisons due to the large variation in change between individual PCCs.

### Meaning of the study: possible mechanisms and implications for clinicians or policy makers

Having the obstacles and challenges of implementing interventions and interpreting data obtained from health care records in mind, our results should be interpreted with some optimism: Clearly, this intervention had an impact on the personnel’s behaviour, seeing that choice of drugs and digit preference altered in line with the recommendations. It resulted in improved cardiovascular risk management with a lowering of LDL-C [32], and for some of the PCC’s included this effect was drastic.

The intervention did not lead to measures against elevated blood pressures that significantly improved blood pressure control compared to a control group. There could be several reasons for these results, e.g. short follow-up time, few PCCs in the intervention and large variations between them. In the end, antihypertensive treatment is a complex affair with multiple choices of drugs and a constantly variable blood pressure value prone to bias and measurement errors, whereas cholesterol treatment often comes down to one single question: to treat or not to treat. It is therefore not surprising that the impact on cholesterol management is more obvious. However, we see indirect signs of influence on the way high blood pressure is managed by distinct change in drug choice, and decrease of digit preference as a sign of impact on measurement procedures.

Many patients still do not achieve the normalized blood pressure they could have had, with a different care. It is important to keep working for a standardized and effective hypertension care, but also to standardize the way blood pressure is measured and registered in the medical journals [31], to minimize sources of error and enhance proper evaluation.

## Conclusions

The results show that our staff training intervention including nurse-led hypertension care had an impact on cholesterol management, digit preference and the choice of antihypertensive drugs, but not on increasing total number of these drugs or reaching blood pressure targets. Compared to cholesterol treatment, blood pressure monitoring and treatment are more complex and prone to measurement errors, with large variations in results between the individual PCCs. Although the intervention failed to improve blood pressure results, it had positive effects on other factors for cardiovascular prevention.
